# Comparative Phosphoproteomics of Neuro-2a Cells under Insulin Resistance Reveals New Molecular Signatures of Alzheimer’s Disease

**DOI:** 10.3390/ijms23021006

**Published:** 2022-01-17

**Authors:** Dayea Kim, Yeon Suk Jo, Han-Seul Jo, Sungwon Bae, Yang Woo Kwon, Yong-Seok Oh, Jong Hyuk Yoon

**Affiliations:** 1 New Drug Development Center, Daegu-Gyeongbuk Medical Innovation Foundation (K-MEDI hub), Daegu 41061, Korea; dayea@kmedihub.re.kr; 2 Neurodegenerative Diseases Research Group, Korea Brain Research Institute, Daegu 41062, Korea; jys0801@kbri.re.kr (Y.S.J.); hanseul@kbri.re.kr (H.-S.J.); maria1101@kbri.re.kr (S.B.); rnjsdiddn@kbri.re.kr (Y.W.K.); 3 Department of Brain-Cognitive Science, Daegu-Gyeongbuk Institute of Science and Technology (DGIST), Daegu 42988, Korea

**Keywords:** phosphoproteomics, insulin resistance, metabolic disease, integrin, AMPK

## Abstract

Insulin in the brain is a well-known critical factor in neuro-development and regulation of adult neurogenesis in the hippocampus. The abnormality of brain insulin signaling is associated with the aging process and altered brain plasticity, and could promote neurodegeneration in the late stage of Alzheimer’s disease (AD). The precise molecular mechanism of the relationship between insulin resistance and AD remains unclear. The development of phosphoproteomics has advanced our knowledge of phosphorylation-mediated signaling networks and could elucidate the molecular mechanisms of certain pathological conditions. Here, we applied a reliable phosphoproteomic approach to Neuro2a (N2a) cells to identify their molecular features under two different insulin-resistant conditions with clinical relevance: inflammation and dyslipidemia. Despite significant difference in overall phosphoproteome profiles, we found molecular signatures and biological pathways in common between two insulin-resistant conditions. These include the integrin and adenosine monophosphate-activated protein kinase pathways, and we further verified these molecular targets by subsequent biochemical analysis. Among them, the phosphorylation levels of acetyl-CoA carboxylase and Src were reduced in the brain from rodent AD model 5xFAD mice. This study provides new molecular signatures for insulin resistance in N2a cells and possible links between the molecular features of insulin resistance and AD.

## 1. Introduction

Neurons are high-energy consuming brain cells [1] that use energy to generate action and postsynaptic potentials and biosynthesize neurotransmitters [2]. Glucose is the primary energy source used by neurons, and its uptake is normally stimulated by insulin [3]. In addition to its general role in energy metabolism, after the late discovery of the insulin receptor, it was revealed that insulin plays a role in the brain by stimulating the hypothalamic satiety center and inhibiting the feeding behavior [4]. It has been reported that the brain’s ability to undergo structural and functional changes in response to environmental stimuli is modulated by insulin. Insulin treatment of hippocampal neurons induces both presynaptic and postsynaptic effects [5]. Insulin increases the basal level of neurotransmitter release from presynaptic terminals by enhancing the frequency of miniature excitatory postsynaptic currents [6]. Insulin also promotes synaptic plasticity by modulating long-term potentiation (LTP) or long-term depression (LTD) in hippocampal synapses through a metaplastic mechanism [7,8]. Insulin administration reduces the stimulation frequency threshold required for inducing LTP and LTD [9]. Accordingly, alterations in insulin signaling in the brain can cause brain aging and regulate brain plasticity; therefore, it could promote neurodegeneration in the late stage of Alzheimer’s disease (AD) [10].

Obesity and/or increased fat intake are associated with cognitive decline and a higher risk of dementia development [11,12]. In obesity-related insulin resistance and type 2 diabetes (T2D), high-fat meals result in excess serum levels of saturated fatty acids (SFAs), which impair metabolism and cause peripheral insulin resistance [13,14]. Palmitate is the most abundant SFA present in the circulation and cerebrospinal fluid [15]. Increased brain uptake and palmitate accumulation have been reported in patients with obesity and metabolic syndrome [16]. In mice, SFAs—including palmitate—have been shown to affect the hypothalamus, a key regulator of peripheral metabolic homeostasis [17]. Palmitate has been related to insulin resistance, endoplasmic reticulum stress, and increased pro-inflammatory responses in the hypothalamus of mice [18,19,20].

Obesity has also been correlated with an increase in low-grade inflammation, which is linked to an increase in tumor necrosis factor-α (TNF-α) and other circulating cytokines [21,22]. Some studies have shown that TNF-α is associated with an increased risk of developing T2D, acting through an intersection of the TNF-α and insulin signaling pathways to induce insulin resistance [23,24]. Insulin resistance is also caused by TNF-α in peripheral tissues, such as the liver, muscle, and adipocytes. Although a wealth of literature has focused on the link between inflammation and insulin resistance in peripheral metabolic tissues, further investigations are required regarding neuroinflammation as a central regulator of energy homeostasis in the hypothalamus [24,25,26,27]. Furthermore, while diabetes is known to increase the risk of dementia, the underlying mechanisms linking insulin resistance, T2D, and AD are poorly understood [28,29].

It has been reported that hippocampal neurogenesis plays a critical role in learning and memory [30,31,32]; its impairment has been associated with cognitive dysfunction in AD [32,33]. Insulin is a well-known key factor in brain development and the control of neurogenesis, including in the hippocampus [34,35]. Indeed, the activation of neuroblasts from quiescence is regulated by activation of the insulin pathway [36,37]. Evidence from in vitro and in vivo experiments indicate that insulin and insulin-like growth factor (IGF)-I promote neurogenesis by modulating neural stem cell (NSC) proliferation, differentiation, and survival [38,39,40]. However, chronic hyperactivation of insulin/IGF-I signaling cascades can cause premature depletion of the NSC reservoir [41]. Thus, insulin may produce either trophic or detrimental effects on the neural stem niche based on the timing and duration of stimulation.

Model animals suffering from insulin resistance (IR) have been reported to display lower preference indexes in the novel object recognition (NOR) test [42,43,44]. Moreover, a recent study model of non-obese T2D performed on Goto-Kakizaki (GK) rats showed that they displayed spatial memory impairment in the Y-maze task and hippocampal synaptic dysfunction [45]. GK rats also showed a reduction in synaptosomal associated protein 25 and synaptophysin levels, suggesting synapse degeneration [46,47]. In addition, insulin receptor substrate 53 knockout mice showed impaired learning and memory in Morris water maze and NOR tests [48,49]. In humans, cerebral glucose metabolism is tightly correlated with neuronal activity [50,51]. Imaging of local brain hypo-metabolism can be used to visualize areas of reduced synaptic activity [52]. Reduced cerebral glucose metabolism is one of the earliest signs of AD [53,54]. Studies in both humans and experimental models suggest that altered brain glucose metabolism is associated with AD progression [55,56]. Furthermore, a recent report provided evidence for the involvement of insulin in amyloid-beta (Aβ) deposition and the AD-dependent impairment of synaptic plasticity and memory formation [57]. It has also been reported that intranasal insulin administration improves cognitive function in humans [58,59]. Taken together, there is much evidence to explain the relationship between insulin resistance and AD, but the precise molecular mechanism is still unclear [41]. It is necessary to clarify the molecular mechanism linking insulin resistance and AD with the exception of insulin itself. Furthermore, given the close relationship between insulin resistance and cognitive decline in AD, the ability of identifying biomarkers capable of detecting brain insulin resistance before—or possibly even in the absence of—peripheral insulin resistance may be predictive of age- and dementia-related cognitive impairment.

The phosphorylation is a type of posttranslational modification of proteins that regulates various aspects of their functionalities [60,61]. Protein phosphorylation plays a key role in cell signaling, gene expression, and differentiation [62,63]. It is also involved in the global control of DNA replication during the cell cycle and in mechanisms that cope with stress-induced replication blocks [64]. Kinases catalyze protein phosphorylation by attaching phosphate groups to specific amino acids [65,66]. In contrast to phosphorylation, dephosphorylation uses phosphatases to remove phosphate groups from proteins. Dephosphorylation plays a vital role in balancing the protein phosphorylation status of signaling proteins [67,68]. Phosphoproteomics is a specific type of proteomics that characterizes proteins with reversible post-translational modification of phosphorylation. Current knowledge on phosphorylation-mediated signaling networks has been dramatically advanced, mainly due to the emerging field of phosphoproteomics [69]. Phosphoproteomics technology has become indispensable for biomedical research; it often enables the quantitative profiling of site-specific phosphorylation across many different biological conditions with extensive phosphoproteome coverage [70]. Furthermore, to the best of our knowledge, there have been no phosphoproteomic studies on brain cells under insulin resistance.

In this study, we applied a reliable phosphoproteomic approach to Neuro-2a (N2a) cells to understand their molecular features under two different clinically reliable insulin-resistant conditions induced by either TNF-α or palmitate. We found different informatic characteristics between the two phosphoproteomes. Through in-depth comparative analysis, we found commonly changed molecular signatures—including molecules in the integrin and adenosine monophosphate-activated protein kinase (AMPK) pathways—and verified them with subsequent biochemical experiments. We finally found that the phosphorylation of acetyl-CoA carboxylase and Src proteins was also altered in the brains of 5xFAD mice. In conclusion, we provide new molecular signatures for insulin resistance in N2a cells according to a comparative phosphoproteomic approach, and ACC and Src are possible molecular signatures to link between insulin resistance and AD.

## 2. Results

### 2.1. Induction of Clinically Reliable Insulin Resistance on N2a Cells

**Figure 1:**
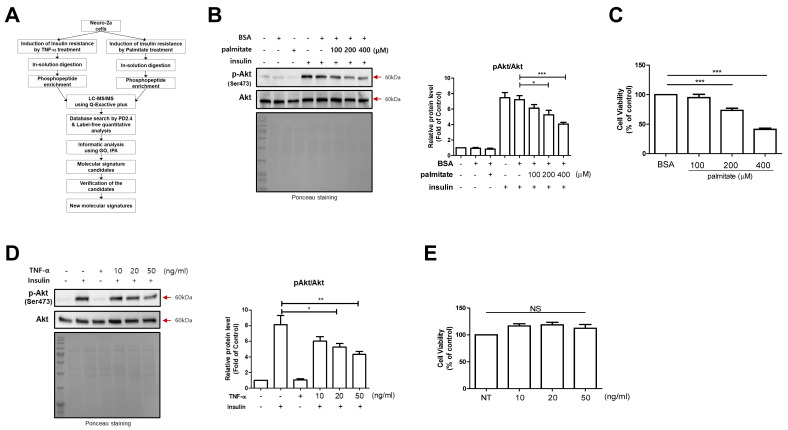
Induction of two different insulin-resistant conditions on Neuro-2a (N2a) cells. (**A**) Experimental workflow. PD 2.4 indicates Proteome Discoverer 2.4, GO indicate gene ontology, and IPA indicates Ingenuity pathway analysis, respectively. (**B**) Western blot of phosphorylated protein kinase B (Akt) that mediated insulin downstream signals under palmitate-induced insulin resistance. Conditioned cell lysates were electrophoresed and blotted. “Insulin” indicates 100 nM of insulin treatment for 10 min. (**C**) The cell viabilities of N2a cells under palmitate treatment. The *y*-axis indicates the relative % change against the bovine serum albumin (BSA)-only condition in the Cell Counting Kit 8 (CCK8) assay. (**D**) Western blot of phosphorylated Akt that mediated insulin downstream signals under tumor necrosis factor (TNF)-α-induced insulin resistance. Conditioned cell lysates were electrophoresed and blotted. “Insulin” indicates 100 nM of insulin treatment for 10 min. (**E**) The cell viabilities of N2a cells under TNF-α treatment. The *y*-axis indicates relative % change against the BSA-only condition in CCK8 assay. All data in the figure are presented as mean ± standard error (SE); *n* = 3–5 per group; * *p* < 0.05, ** *p* < 0.005, *** *p* < 0.0005.

Our experimental strategy for the research is outlined in Figure 1A. To induce clinically reliable insulin resistance in N2a cells, we used palmitate and TNF-α to mimic dyslipidemia and inflammation, respectively [71,72]. In palmitate-induced insulin resistance, we treated the cells with palmitate in a dose-dependent manner and determined the phosphorylation of protein kinase B (Akt) on S473 by Western blotting. We found a gradual decrease in the insulin-induced S473 phosphorylation level of Akt following palmitate treatment (Figure 1B). After measuring cell viability, we selected 200 μM as the optimal concentration for the insulin-resistant condition (Figure 1C). TNF-α-induced insulin resistance was also tested on N2a cells in the same manner as in the palmitate experiment. After measuring cell viability, we selected 20 ng/mL of TNF-α as the optimal concentration for insulin resistance (Figure 1D,E).

### 2.2. Phosphoproteomic Analysis Using Reliable Proteomics Approach

**Figure 2:**
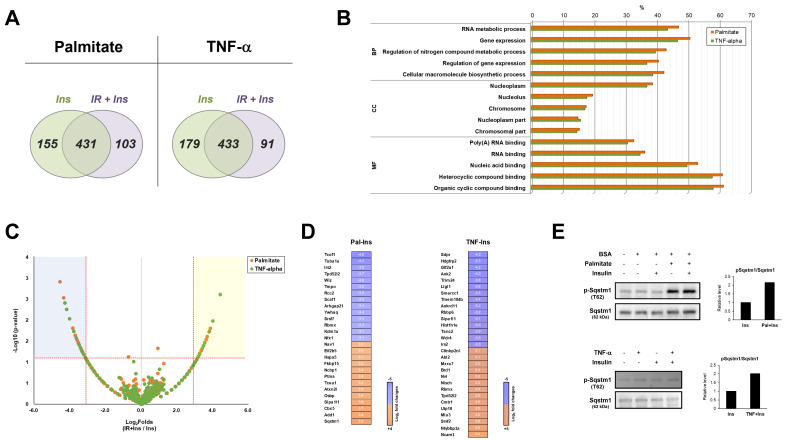
Phosphoproteomics of two different insulin-resistant conditions. (**A**) Venn diagram of the identified proteins. “Ins” and “IR + Ins” indicate the insulin-treated condition and insulin resistance then insulin-treated conditions, respectively. (**B**) Gene ontology (GO) enrichment analysis of two independent phosphoproteomes. “BP”, “CC”, and “MF” indicate biological process, cellular component, and molecular function, respectively. Significantly enriched GO terms are shown with the Benjamini–Hochberg false discovery rate-corrected percentage. (**C**) Volcano plot of significantly changed proteins of two independent phosphoproteomes. The −log_10_ (*p*-value) is plotted against the log_2_ (fold change: IR + Ins/INS). The non-axial vertical lines denote a ±3-fold change, whereas the non-axial horizontal line denotes *p* = 0.05, which is the significance threshold (prior to logarithmic transformation). (**D**) Heatmap representing the log_2_ fold changes of significantly changed phosphoproteins under each insulin-resistance condition. The decreased and increased phosphoproteins are shown in blue and orange, respectively. (**E**) Western blot of the significantly changed phosphoproteins under each insulin-resistant condition. Conditioned cell lysates were electrophoresed and blotted. “Insulin” indicates 100 nM of insulin treatment for 10 min.

For phosphoproteomic analysis, we used N2a cell lysates from two different insulin-resistant conditions induced by palmitate and TNF-α. We used optimized in-solution digestion for peptide generation. The peptides from the cell lysates were subjected to electrospray ionization (ESI)–tandem mass spectrometry (MS/MS). We searched the UniProt database using the Sequest search engine featured in Proteome Discoverer 2.4. The resulting phosphoproteins were quantitatively analyzed using spectral count-based label-free quantitative analysis. We used phosphoproteomes both treated and untreated with insulin resistance. Insulin is commonly used to induce insulin-dependent phosphorylation. In the palmitate condition, we identified 689 phosphoproteins from the insulin treatment (Ins) and insulin resistance–insulin treatment (IR + Ins) groups (Table S1). A total of 155 proteins and 103 phosphoproteins were exclusively identified in the Ins and IR + Ins groups, respectively. A total of 431 proteins were identified (Figure 2A). In the TNF-α condition, we identified 703 phosphoproteins from the Ins and IR + Ins groups (Table S1). A total of 179 proteins and 91 phosphoproteins were exclusively identified in Ins and IR + Ins, respectively. A total of 433 proteins were identified (Figure 2A).

To identify the biological features of the two different insulin resistances, we first used database for annotation, visualization, and integrated discovery (DAVID) bioinformatics tools for functional annotation. For the palmitate phosphoproteome, the enrichment analysis by gene ontology biological processes (GOBPs) revealed that the phosphoproteins were involved in RNA metabolic processes, gene expression, regulation of nitrogen compound metabolic processes, regulation of gene expression, and the cellular macromolecule biosynthetic process. The analysis of gene ontology cellular components (GOCCs) identified nucleus-related terms, such as nucleoplasm, nucleolus, and chromosome. In gene ontology molecular function (GOMF) analysis, the phosphoproteins were found to be involved in poly(A) RNA binding, RNA binding, nucleic acid binding, and compound binding. Interestingly, we found that the GO terms and percentage of the TNF-α phosphoproteome were highly similar to those of palmitate (Figure 2B).

To visualize significantly changed proteins, volcano plots were performed using the two proteomes (Figure 2C). Points above the non-axial horizontal line represent proteins with significantly different abundances (*p* < 0.05). The points to the left of the left-most non-axial vertical line denote protein fold changes of the IR + Ins group versus the Ins group that are less than −3, while points to the right of the right-most non-axial vertical line denote protein fold changes of the IR + Ins group/Ins group that are greater than 3. When we compared the phosphoproteins most affected by insulin resistance in the criteria with ≥|3| log_2_ fold changes, there was little similarity. Four proteins were recapitulated: insulin receptor substrate 2 (Irs2), RNA-binding motif protein (Rbmx), signal-induced proliferation-associated 1-like protein 1 (Sipi1l1), and tumor protein D54 (Tpd52l2). However, only phosphorylation of insulin receptor substrate 2 was commonly decreased under both insulin-resistant conditions (Figure 2D). Lists of the significantly changed phosphoproteins under the two different insulin-resistant conditions are shown in Table 1 and Table 2 with the identified modification sites. Next, we performed Western blotting to verify this observation. We found that phosphorylation of sequestosome-1 was clearly increased under palmitate-induced insulin resistance, compared to the slight increase in TNF-a-induced insulin resistance (Figure 2E). Taken together, we found that functional annotation by GO showed a highly similar pattern between the two phosphoproteomes. However, there is quite a difference at the molecular level, especially in the most significantly changed phosphoproteins.

### 2.3. Informatics Analysis of Each Phosphoproteome

**Figure 3:**
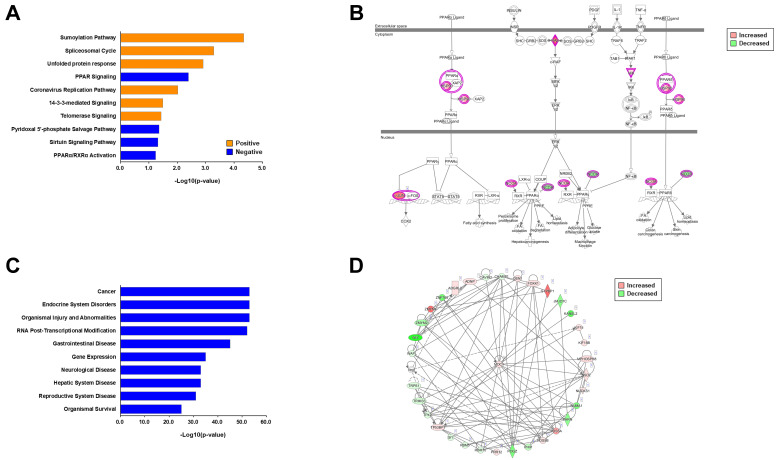
Informatic analysis of phosphoproteomes of palmitate-induced insulin-resistant conditions. (**A**) Canonical pathway enrichment analysis of the 689 phosphoproteins. The positive and negative z-scores are shown in orange and blue color, respectively. The *x*-axis indicates the −log10 (*p*-value) of each pathway. (**B**) The peroxisome proliferator-activated receptor (PPAR) pathway as the representative canonical pathway of phosphoproteomes of palmitate-induced insulin-resistant conditions. The increased and decreased phosphorylation levels are shown in red and green, respectively. (**C**) Top ten terms of disease relation and biological functions. The *x*-axis indicates the −log10 (*p*-value) of each term. (**D**) Interactome analysis using the top1 term (cancer)-related phosphoproteins. The increased and decreased phosphorylation levels are shown in red and green, respectively.

Next, we performed an in-depth informatic analysis for each phosphoproteome to understand different insulin-resistant conditions in neurons. For the phosphoproteome by palmitate-induced insulin resistance, we first performed canonical pathway analysis using ingenuity pathway analysis (IPA). We found that six canonical pathways were upregulated (Z-score > 0) and four canonical pathways were downregulated (Z-score < 0) (Figure 3A). We found that the sumoylation pathway was the most significantly changed canonical pathway by palmitate-induced insulin resistance. Because insulin resistance is majorly linked to the pathological responses of cellular pathways, we mainly focused on the negatively correlated canonical pathways. Peroxisome proliferator-activated receptor (PPAR) signaling was found to be the most downregulated [−log_10_ (*p*-value) = 4.34] pathway. The PPAR signaling pathway includes diverse phosphoproteins, such as heat shock proteins, c-Jun, Ras, and nuclear receptor corepressor/coactivator. These phosphoproteins participated in the PPAR signaling pathway to inhibit the activation pathway by different phosphorylation mechanisms (Figure 3B). We then surveyed the disease relations and biological functions. We found that cancer, endocrine system disorders, organismal injury, and abnormalities were the most significantly related diseases (Figure 3C). Because there are various cancers and relatively well-curated bioinformatics information on it, it is necessary to focus on molecular scales—such as interactomes—to discriminate sophisticated differences. Interactome analysis using the cancer-related phosphoproteome of palmitate-induced insulin resistance revealed that several proteins interacted with each other, and mediator of DNA damage checkpoint protein 1 (MDC1) was the central protein in cancer signal transduction (Figure 3D).

**Figure 4:**
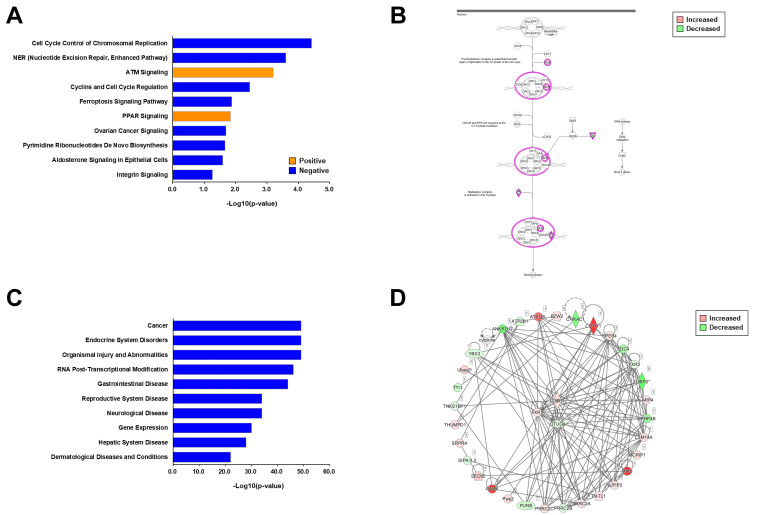
Informatic analysis of phosphoproteomes of TNF-α-induced insulin-resistant conditions. (**A**) Canonical pathway enrichment analysis of the 703 phosphoproteins. The positive and negative z-scores are shown in orange and blue, respectively. The *x*-axis indicates the −log10 (*p*-value) of each pathway. (**B**) The cell cycle control of chromosomal replication pathway as the representative canonical pathway of phosphoproteomes of TNF-α-induced insulin-resistant conditions. The increased and decreased phosphorylation levels are shown in red and green, respectively. (**C**) Top 10 terms of disease relation and biological functions. The *x*-axis indicates the −log10 (*p*-value) of each term. (**D**) Interactome analysis using the Top 10 terms of (cancer)-related phosphoproteins. The increased and decreased phosphorylation levels are shown in red and green, respectively.

When we analyzed the phosphoproteome by TNF-α-induced insulin resistance, it was found that two canonical pathways were upregulated and eight canonical pathways were downregulated (Figure 4A). We also found that the cell cycle control of chromosomal replication was the most significantly changed and downregulated canonical pathway [−log_10_ (*p*-value) = 4.42] with the involvement of diverse phosphoproteins such as Cdks, Mcms, and Top2s. These phosphoproteins participated in the signaling pathway to inhibit the activation pathway by different phosphorylation (Figure 4B). Next, we also surveyed the disease relations and biological functions of the TNF-α phosphoproteome. We found that cancer, endocrine system disorders, organismal injury, and abnormalities were the most significantly related diseases (Figure 4C), which was the same as with the palmitate phosphoproteome. We then checked the interactome of the top-related disease (cancer) to determine whether the interactome was similar to that for palmitate phosphoproteome. Interestingly, it showed different interactions and the centered proteins were Ras GTPase-activating protein-binding protein 1 (G3bp1), fragile X mental retardation syndrome-related protein 1 (Fxr1), and OTU domain-containing protein 4 (Otud4) (Figure 4D). In conclusion, there were clearly different pathways in canonical pathway analysis between the two different phosphoproteomes. Although the most related diseases were recapitulated, the interactome and centered proteins were quite different between the two phosphoproteomes. These results indicate that different phosphorylation patterns occur according to the insulin-resistant condition.

### 2.4. Comparative Informatics Analysis for Two Phosphoproteomes

**Figure 5:**
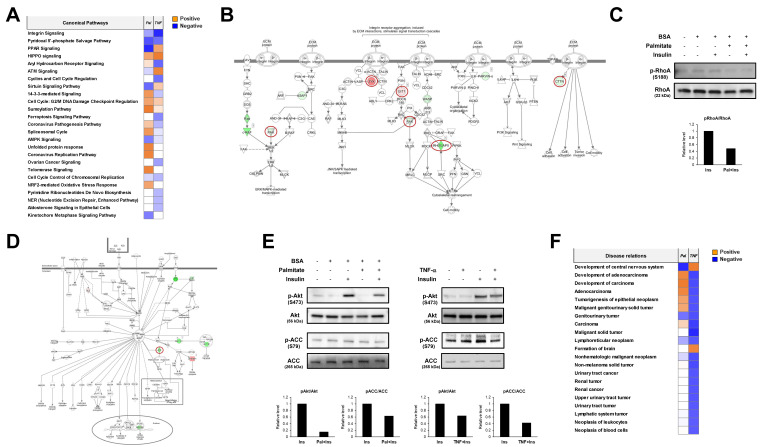
Comparative informatic analysis of phosphoproteomes of two different insulin-resistant conditions. (**A**) Comparative canonical pathway analysis between the phosphoproteomes. The positive and negative z-scores are shown in orange and blue, respectively. “Pal” and “TNF” indicate palmitate-induced insulin-resistant and TNF-α-induced insulin-resistant conditions, respectively. (**B**) The integrin pathway was the most downregulated pathway between the phosphoproteomes. The increased and decreased phosphorylation levels are shown in red and green, respectively. The red circles indicate the phosphoproteins commonly regulated by different insulin-resistant conditions. (**C**) Western blot of phospho-RhoA under palmitate-induced insulin-resistant conditions. Conditioned cell lysates were electrophoresed and blotted. “Insulin” indicates 100 nM of insulin treatment for 10 min. (**D**) The adenosine monophosphate-activated protein kinase pathway was the most downregulated pathway between the phosphoproteomes. (**E**) Western blot of phospho-protein kinase B and phospho-acetyl-CoA carboxylase under different insulin-resistant conditions. Conditioned cell lysates were electrophoresed and blotted. “Insulin” indicates 100 nM of insulin treatment for 10 min. (**F**) Comparative analysis for disease relation and biological functions. The positive and negative z-scores are shown in orange and blue color, respectively. “Pal” and “TNF” indicate palmitate-induced insulin-resistant condition and TNF-α-induced insulin-resistant condition, respectively.

To determine the common molecular signatures under different insulin resistance conditions, we carried out comparative informatic analysis of both phosphoproteomes on the same line. In comparative canonical pathway analysis, we found the opposite direction in regulation mode for six pathways, comprising PPAR signaling, aryl hydrocarbon receptor signaling, ATM signaling, sirtuin signaling, 14-3-3-mediated signaling, and spliceosomal cycle (Figure 5A). Seven pathways were involved in the regulation mode, comprising integrin signaling, pyridoxal 5′-phosphate salvage pathway, HIPPO signaling, cell cycle, sumoylation pathway, AMPK signaling, and chromosomal replication (Figure 5A). Among the canonical pathways observed in the negative direction in regulation mode, we focused on two canonical pathways: integrin signaling as the most significantly changed pathway, and AMPK signaling as a key metabolic regulation pathway.

In the molecular pathway of integrin signaling, six proteins were commonly regulated by different insulin-resistant conditions. Serine/threonine-protein kinase Pak, Rho GTPase activating protein 5 (Rhogap5), and cortactin (Cttn) showed decreased phosphorylation levels under two insulin-resistant conditions (Figure 5B). Zyxin (Zyx) and the ARF GTPase-activating protein Git increased their phosphorylation levels under insulin-resistant conditions. We then tried to verify whether the integrin signaling pathway is modulated by insulin resistance. Because most of the identified phosphosites of these proteins have not yet been studied well, we checked the activity of upstream kinases—such as RAC1/Cdc42, RhoA, and Src—which are upstream kinases for Pak, Cttn, and Rhogap5. In Western blotting analysis using N2a cells under insulin resistance, we found that phosphorylation of RAC/Cdc42 was commonly decreased under both insulin-resistant conditions (Figure 5C). The phosphorylation of RhoA was decreased in palmitate-induced insulin resistance, but was only marginally decreased in TNF-α induced insulin resistance. From these results, we concluded that the integrin pathway of N2a cells is downregulated through modulation of the common molecular targets under palmitate-induced and TNF-α-induced insulin resistance.

In the molecular pathway of AMPK signaling, acetyl-CoA carboxylase (ACC) was found to be downregulated by different insulin-resistant conditions (Figure 5D). We also tried to verify whether the AMPK signaling pathway is modulated by insulin-resistant conditions. In Western blotting analysis, we found that phosphorylation of AMPK was increased in both insulin-resistant conditions (Figure 5E). ACC phosphorylation was decreased in palmitate-induced and TNF-induced insulin resistance. We found that the AMPK pathway of N2a cells is regulated through modulation of the common molecular targets under palmitate- and TNF-α-induced insulin resistance. Comparative analysis of diseases and biological functions revealed cancer-related diseases and functions. We found the opposite relationship for each related disease for palmitate and TNF-α-induced insulin resistance conditions (Figure 5F). Taken together, we found that there are different patterns in canonical pathways and disease relationships between the phosphoproteomes of palmitate- and TNFα-induced insulin resistance. More importantly, we conclude that both integrin signaling and AMPK signaling are new molecular signatures of insulin resistance in N2a cells.

### 2.5. Changes of Phosphoproteins in 5xFAD Mice Brain

**Figure 6:**
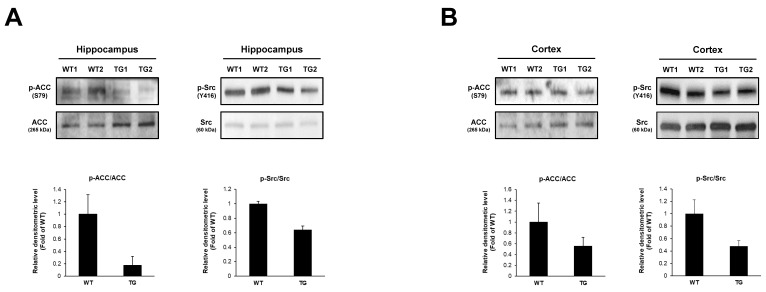
Western blots for phosphorylation of the proteins in 5xFAD mice brain. (**A**) Western blot of phosphorylation of acetyl-CoA carboxylase and Src in the hippocampus of 5xFAD mice brains. (**B**) Western blot of phosphorylation of acetyl-CoA carboxylase and Src in the cortex of 5xFAD mice brains. Tissue lysates were electrophoresed and blotted. WT and TG indicate wild-type and 5xFAD mice, respectively.

To find a possible correlation between the insulin resistance of neurons and neurodegenerative disease, we performed Western blotting analysis for the key phosphoproteins involved in AMPK and integrin pathways in the brains of 5xFAD mice. We found that the phosphorylation of ACC was decreased in both the hippocampus and cortex of 5xFAD mice (Figure 6A). We also found that the phosphorylation of Src was decreased in both the hippocampus and cortex of 5xFAD mice (Figure 6B). These results indicate a possible relationship between the phospho-signaling pathway and insulin resistance in neurons and AD.

## 3. Discussion

In this study, we used a phosphoproteomic approach on N2a cells to understand the molecular features under two different insulin-resistant conditions induced by either TNF-α or palmitate. Through comparative bioinformatics analysis, we characterized the similarity and difference between these two insulin-resistant phosphoproteomes. Func-tional annotation of GO showed a similar pattern but was quite different at the molecular level, especially in the most significantly changed phosphoproteins. In canonical pathway analysis, the PPAR signaling pathway was most downregulated under palmitate-induced insulin resistance. The cell cycle control of the chromosomal replication pathway was the most downregulated canonical pathway in TNF-α-induced insulin resistance. Both phosphoproteomes shared the same target diseases in the disease relations—including cancer, endocrine system disorders, organismal injury, and abnormalities—with high statistical significance; however, the interactomes and centered proteins were quite different between the two phosphoproteomes. Based on these results, different phosphorylation patterns occurred according to the given insulin-resistant condition in N2a cells. In comparative canonical pathway analysis between two phosphoproteomes, we found the op-posite direction—such as PPAR signaling, aryl hydrocarbon receptor signaling, ATM sig-naling, sirtuin signaling, 14-3-3-mediated signaling, and spliceosomal cycle; and the same direction—such as integrin signaling, pyridoxal 5′-phosphate salvage pathway, HIPPO signaling, cell cycle, sumoylation pathway, AMPK signaling, and chromosomal replication. Next, we further studied two canonical pathways, including integrin signaling, as the most significantly changed pathway, and AMPK signaling as a key metabolic regulation pathway. Finally, we checked the possible link between the molecular features of insulin resistance and AD.

In comparative pathway analysis, we found important information about the existence of the same and opposite directed canonical pathways between the two different phosphoproteomes. The opposite directed canonical pathways indicate that these pathways are regulated differently, such as in the activation and inhibition by each of the insulin-resistant conditions. It is expected that this finding will elucidate the precise molecular mechanism of each insulin-resistant condition in neurons. It is essential to conduct an in-depth study of each insulin-resistant condition in neurons. However, the information of opposite-directed canonical pathways will be helpful in the characterization of each related disease and their relation to brain functions. The same directed canonical pathways indicate that these pathways are regulated in the same way, such as activation or inhibition by each of the insulin-resistant conditions. Unlike the oppositely directed pathways, the same directed canonical pathways provide more valuable information. The same directed canonical pathways share the same molecular mechanisms, even though there are different causes of insulin resistance. This means that there are molecular target candidates that regulate neuronal pathology under insulin resistance, such as neurodegenerative diseases and psychiatric disorders. We also focused on the same directed canonical pathways to identify common molecular events under different insulin-resistant conditions. Although not perfect, we found and verified the integrin and AMPK pathways as those kinds of pathways. It is possible that comparative pathway analysis can help understand the molecular features of different pathological conditions and discover important molecular signatures in diseases.

Integrin is a receptor composed of α and β transmembrane subunit heterodimers which induce intracellular responses by binding to the extracellular matrix or ligands. Integrin signaling plays a vital role in corticogenesis through the regulation of neurogenesis [73]. Many studies have shown an association between integrin and AD [74]. Aβ modulates α1β1 integrin in neuronal cells, whereas estradiol abolishes downregulated integrin expression [75]. In contrast, the integrin subunit αv mediates Aβ peptide-induced LTP inhibition, which affects neuronal plasticity [76] and increased β2 integrin in the AD brain is involved in Aβ-induced neuroinflammation [77]. A recent study revealed that the αv/β1 integrin complex acts as a receptor for Tau monomers and fibrils in primary astrocytes, mediating the internalization of tau fibrils and activating integrin signaling, finally inducing inflammatory responses in astrocytes and contributing to the tau pathology mechanism [78]. In this study, we found that the integrin pathway is deactivated by palmitate-induced insulin resistance. AD mouse brains also showed deactivated integrin pathways. Therefore, it is necessary to elucidate the direct relationship between the integrin pathway and AD pathology.

AMPK is activated by AMP or ADP, which are increased during energy depletion in cells and play a crucial role in maintaining energy homeostasis by inducing glucose uptake and fatty acid oxidation [79]. Many neurodegenerative diseases are known to be accompanied by energy metabolism disorders [80]. The activation of AMPK increases the phosphorylation of ACC, decreases the expression of fatty acid synthase, and prevents oxidative stress and inflammatory responses in the brains of high cholesterol-fed mice [81]. AMPK agonists ameliorate impaired insulin sensitivity and improve the molecular and pathological features of AD [82]. It is known that Aβ oligomers temporarily inhibit AMPK and cause metabolic dysfunction in hippocampal neurons in the early AD brain [83]. In addition, AMPK alleviates AD by activating sirtuin 1, whose expression is reduced with impairment of spatial learning and memory [84,85]. Previous studies have confirmed the association between integrin or AMPK and neurodegenerative disease, and our results showed changes in the integrin and AMPK pathways in insulin resistance-induced neuronal cells, suggesting the possibility that metabolic disease may induce AD. Therefore, the results of this study need to be further verified by confirming whether there is any relevance to the pathological progression of insulin resistance-induced AD mice and whether there is an improvement effect by regulating the integrin or AMPK signaling pathway.

Collectively, we here profiled prophopreoteome features under two different insulin-resistant conditions with clinical relevance: inflammation and dyslipidemia. Despite significant difference in overall phosphoproteome profiles, we found molecular signatures and biological pathways in common between two insulin-resistant conditions. Among various phosphoproteome changes upon insulin resistance induction, we found that the phosphorylation levels of acetyl-CoA car-boxylase and Src are reduced also in the brains of rodent AD model 5xFAD mice. Our study identified new molecular signatures for insulin resistance which provide possible links between insulin resistance and AD.

## 4. Materials and Methods

### 4.1. Animal Management

All experimental procedures involving animals were approved by the Korea Brain Research Institute Animal Use and Care Committee. TG6799 mice were kept under a 12-h light/dark cycle with free access to water.

### 4.2. Establishment of Two Different Insulin Resistance Conditions

Sodium-palmitate (Sigma-Aldrich, St. Louis, MO, USA) was dissolved in ethanol and diluted in 10% fatty acid-free bovine serum albumin (BSA). The mixture was then heated to 60 °C for 30 min. To induce palmitate-mediated insulin resistance, N2a cells were incubated with 200 μM of palmitate-BSA mixture solution for 16.5 h in culture medium supplemented with 10% FBS. N2a cells were incubated with the same concentration of palmitate-BSA mixture solution in serum-free medium for 1.5 h. To induce TNF-α-mediated insulin resistance, N2a cells were incubated with 20 ng/mL of TNF-α (R&D Systems, Minneapolis, MN, USA) for 24 h. After incubation, N2a cells were washed three times with phosphate-buffered saline (PBS) and incubated in culture medium supplemented with 0.1% BSA in the absence of TNF-α for 90 min. After treatment with each insulin-resistant condition, N2a cells were treated with 100 nM insulin for 10 min.

### 4.3. Phosphopeptide Preparation

For in-solution digestion, N2a cells were dissolved in 0.2% ProteaseMAX (V2071; Promega, Madison, WI, USA) in 40 mM ammonium bicarbonate (NH_4_HCO_3_). After sonication, the cell lysates were 4-fold diluted with 40 mM ABC. After 20 min incubation with 10 mM dithiothreitol at 56 °C, 20 mM iodoacetamide was added and incubated for 20 min at room temperature in the dark. After the bicinchoninic acid (BCA) protein assay, a 1:50 ratio of trypsin Lys-C mixture (V5073; Promega) was added to 100 µg of protein for 4 h at 50 °C. After centrifugation at 16,000× *g* for 10 s at 4 °C, the supernatant was collected. The cells were treated with 0.5% trifluoroacetic acid (28901; Thermo Fisher Scientific, San Jose, CA, USA) for 5 min at 25 °C to stop the reaction. Phosphopeptide enrichment was performed using a TiO_2_ Phosphopeptide Enrichment Kit (A32993; Thermo Fisher Scientific, Waltham, MA, USA) following the manufacturer’s protocol.

### 4.4. Mass Analysis and Database Searching

Tryptic digested phosphopeptides were analyzed using a Q Exactive^TM^ Plus Hybrid Quadrupole–Orbitrap Mass Spectrometer interfaced with an EASY-Spray^TM^ source (Thermo Fisher Scientific). Chromatographic separation of peptides was achieved using an UltiMate^TM^ 3000 RSLCnano system (Thermo Fisher Scientific), equipped with an Acclaim™ PepMap™ 100 C18 HPLC Column (75 μm × 2 cm, 3 μm nanoviper; Thermo Fisher Scientific) as the loading column and an EASY-Spray PepMap RSLC C18 Column (75 μm × 50 cm, 2 μm; Thermo Fisher Scientific) as the separation column. Peptides were loaded from the RS auto-sampler and separated with a linear gradient of acetonitrile (ACN)/water, containing 0.1% formic acid, at a flow rate of 300 nL/min. The liquid chromatography eluent was electrosprayed directly from the analytical column, and a voltage of 2.0 kV was applied via the liquid junction of the nanospray source. Peptide mixtures were separated with a gradient of 10–50% ACN for 80 min. The analysis method consisted of a full MS scan with a range of 350–2000 *m*/*z* and data-dependent MS/MS on the 10 most intense ions from the full MS scan. The mass spectrometer was programmed to the data-dependent acquisition mode. Mass spectrometer calibration was performed using the proposed calibration solution, according to the manufacturer’s instructions.

To perform the database search, tandem mass spectra were processed using Proteome Discoverer software version 2.4 (Thermo Fisher Scientific). The spectral data were searched against the Human Uniprot database (release version 2021_03). All identified proteins had a false discovery rate of ≤1%, which was calculated at the peptide level. Search parameters allowed for a tryptic specificity of up to two missed cleavages, with methylthio-modifications of cysteine as a fixed modification and oxidation of methionine as a dynamic modification. The mass search parameters for +1, +2, and +3 ions included mass error tolerances of 20 ppm for precursor ions and 0.6 Da for fragment ions.

### 4.5. Bioinformatics

DAVID bioinformatics resource 6.8 was used for GO-based function annotation. IPA was used for in-depth bioinformatics analysis. For the identified proteins, Uniprot protein accession numbers coupled with the value of normalized fold changes were uploaded to IPA in the protein expression criteria. We used the following criteria for quantitative pathway analysis: z-score cutoff = 0.5, −log (*p*-value) > 1.3.

### 4.6. Western Blotting

Cells and brain tissues were washed with ice-cold PBS and homogenized using RIPA buffer containing 1× Halt protease and phosphatase inhibitor cocktail (Thermo Fisher Scientific). The homogenate was sonicated, and cellular debris was removed by centrifugation at 13,000× *g* for 15 min at 4 °C. After the determination of protein concentration by BCA protein assay, lysates were mixed with sodium dodecyl sulphate sample buffer. After protein separation by sodium dodecyl sulphate–polyacrylamide gel electrophoresis, the proteins were transferred to poly(vinylidene fluoride) membranes using the Bio-Rad wet transfer system, blocked with PBS-T containing 5% skim milk for 30 min, and then incubated with the antibodies for 16 h at 4 °C. After washing the membranes three times with PBS-T, the blots were incubated with horseradish peroxidase-conjugated anti-mouse or anti-rabbit antibodies for 1 h at room temperature. The membranes were then washed with PBS-T and developed using enhanced chemiluminescence. We performed Western blotting with antibodies against phospho-Sqstm1 (13121s; Cell Signaling Technology, Danvers, MA, USA), Sqstm1 (5144s; Cell Signaling Technology), phospho-RhoA (AF8020, Affinity Biosciences, Cincinnati, OH, USA), RhoA (AF6352, Affinity Biosciences), phospho-ACC (3661s; Cell Signaling Technology), ACC (3662S; Cell Signaling Technology), phospho-Akt (9271s; Cell Signaling Technology), Akt (9272s; Cell Signaling Technology), phospho-AMPK (2535s; Cell Signaling Technology), AMPK (2532s; Cell Signaling Technology).

### 4.7. Cell Viability Assay

Cell viability was measured using the Cell Counting Kit-8 (CCK-8, Dojindo, Kumamoto, Japan) according to the manufacturer’s instructions. Briefly, for the standard curve, N2a cells were serially diluted from 5 × 10^4^ cells. To measure cellular toxicity by TNF-α and BSA-conjugated palmitate, 2.5 × 10^4^ cells/well were plated in 96-well plates and incubated overnight. The medium was replaced with serum-free media to treat TNF-α and BSA-conjugated palmitate and incubated for 24 and 18 h, respectively. After treatment, the cells were washed twice with serum-free medium. Serum-free medium and CCK-8 were mixed at a ratio of 10:1 and treated with 100 µL in each well. CCK-8-treated cells were incubated for 30 min at 37 °C. Cell viability was measured at 450 nm using a microplate reader. The number of viable cells was substituted into a standard curve to calculate the results. The results were expressed as a percentage of the control cells.

### 4.8. Statistical Analysis

All data are expressed as the mean ± SEM. All statistical analyses were performed using Student’s *t*-tests and one-way analysis of variance. Statistical significance was set at *p* < 0.05.

## Figures and Tables

**Table 1:** Significantly changed phosphoproteomes according to palmitate-induced insulin-resistant conditions.

Accession	Gene Symbol	Description	Log_2_Fold (Pal + Ins/Ins)	Modifications
Q64337	*Sqstm1*	Sequestosome-1	3.86	Phospho [T269(100); T271(99.5); S330(100); S334(100); S363(98.7); S367(98.7); S368(98.7)]
Q9QYC0	*Add1*	Alpha-adducin	3.80	Phospho [T610(99.1); T614(99.1); S724(100)]
Q61686	*Cbx5*	Chromobox protein homolog 5	3.69	Phospho [S14(99.6)]
Q8C0T5	*Sipa1l1*	Signal-induced proliferation-associated 1-like protein 1	3.63	Phospho [S1528(100); S1624(100); S1626(100)]
P26350	*Ptma*	Prothymosin alpha	3.35	Met−loss + Acetyl [N-Term]; Phospho [S2(100)]; Acetyl [N-Term]
P10711	*Tcea1*	Transcription elongation factor A protein 1	3.35	Phospho [S100(100)]
Q7TQH0	*Atxn2l*	Ataxin-2-like protein	3.35	Phospho [S109(100); S304(100); S337(99.4)]
Q3B7Z2	*Osbp*	Oxysterol-binding protein 1	3.35	Phospho [S188(100); S191(100); T375(100); S377(99.5); S380(100); S383(100)]
Q3UYV9	*Ncbp1*	Nuclear cap-binding protein subunit 1	3.27	Phospho [S22(99.6)]
Q6P9Q6	*Fkbp15*	FK506-binding protein 15	3.19	Phospho [S1157(100); S1159(100)]
P20029	*Hspa5*	Endoplasmic reticulum chaperone BiP	3.10	Phospho [S650(98)]
Q8CH77	*Nav1*	Neuron navigator 1	3.01	Phospho [S1247(99.2)]
Q8CHW4	*Eif2b5*	Translation initiation factor eIF-2B subunit epsilon	3.01	Phospho [S540(100)]
B1AY10	*Nfx1*	Transcriptional repressor NF-X1	−3.07	Phospho [S51(98.7); S81(100); S147(100); S149(98.6)]
Q6ZQ88	*Kdm1a*	Lysine-specific histone demethylase 1A	−3.14	Phospho [S132(100); S138(100); S167(100)]
Q9WV02	*Rbmx*	RNA-binding motif protein, X chromosome	−3.29	Phospho [S208(100)]
Q6DFV3	*Arhgap21*	Rho GTPase-activating protein 21	−3.36	Phospho [S874(100); T1621(100); S1623(100)]
P68254	*Ywhaq*	14-3-3 protein theta	−3.36	Phospho [S230(96)]
Q8BL97	*Srsf7*	Serine/arginine-rich splicing factor 7	−3.36	Phospho [S208(100); S210(100)]
Q5U4C3	*Scaf1*	Splicing factor, arginine/serine-rich 19	−3.48	Phospho [S510(100); S518(99.5); S676(100); S682(100); S691(100); S695(100); S821(100)]
Q61029	*Tmpo*	Lamina-associated polypeptide 2, isoforms beta/delta/epsilon/gamma	−3.54	Phospho [S66(100); S67(100); T74(100); T159(98.6); S179(100); S183(100)]
Q8BK67	*Rcc2*	Protein RCC2	−3.54	Phospho [S48(95.5)]
O88286	*Wiz*	Protein Wiz	−3.60	Phospho [S1045(100); S1050(100)]
Q9CYZ2	*Tpd52l2*	Tumor protein D54	−3.70	Phospho [S200(99)]
P81122	*Irs2*	Insulin receptor substrate 2	−3.85	Phospho [T55(99); S66(100); T517(100); T524(100); S556(100); S573(100); S616(99.5); S1089(100)]
P68369	*Tuba1a*	Tubulin alpha-1A chain	−4.36	Phospho [S439(100)]
O08784	*Tcof1*	Treacle protein	−4.57	Phospho [S83(97); T1114(99.2); S1191(100)]

**Table 2:** Significantly changed phosphoproteomes according to tumor necrosis factor (TNF)-α-induced insulin-resistant conditions.

Accession	Gene Symbol	Description	Log_2_Fold (TNF + Ins/Ins)	Modifications
P13595	*Ncam1*	Neural cell adhesion molecule 1	4.41	Phospho [S770(100); S774(100); S1005(100)]
Q7TPV4	*Mybbp1a*	Myb-binding protein 1A	3.99	Phospho [S1164(100); S1253(99.1); T1256(99.1); S1280(100)]
Q62093	*Srsf2*	Serine/arginine-rich splicing factor 2	3.79	Phospho [T25(99.4); S206(100); S208(100); S212(100)]
Q5SSI6	*Utp18*	U3 small nucleolar RNA-associated protein 18 homolog	3.55	Phospho [S114(100); S115(100); S118(100); S206(100)]
Q8BI84	*Mia3*	Transport and Golgi organization protein 1 homolog	3.55	Phospho [S1458(100); S1765(99.4)]
Q9CYZ2	*Tpd52l2*	Tumor protein D54	3.48	Phospho [S200(100)]
Q9DBC3	*Cmtr1*	Cap-specific mRNA (nucleoside-2′-O-)-methyltransferase 1	3.48	Phospho [S27(99.4); S48(98.6); S50(100); S52(100); S54(100)]
Q9WV02	*Rbmx*	RNA-binding motif protein, X chromosome	3.41	Phospho [S208(100)]
G5E8P1	*Brd1*	Bromodomain-containing protein 1	3.26	Phospho [S128(100); S1052(100); S1055(100)]
Q9DBY8	*Nvl*	Nuclear valosin-containing protein-like	3.26	Phospho [S190(100)]
Q80TM9	*Nisch*	Nischarin	3.26	Phospho [S543(99.5); S548(99.7); S1373(100)]
Q9CZH7	*Mxra7*	Matrix-remodeling-associated protein 7	3.18	Phospho [S79(100)]
Q99LJ0	*Cttnbp2nl*	CTTNBP2 N-terminal-like protein	3.09	Phospho [S481(100); S556(100); S559(100); S562(100)]
Q4JIM5	*Abl2*	Tyrosine-protein kinase ABL2	3.09	Phospho [S621(100); S632(99.5)]
P43274	*Hist1h1e*	Histone H1.4	−3.03	Met−loss + Acetyl [N-Term]; Phospho [S2(99.2); T18(100)]; Acetyl [N-Term]
A2A690	*Tanc2*	Protein TANC2	−3.03	Phospho [S1534(100); S1538(100)]
Q9EP82	*Wdr4*	tRNA (guanine-N(7)-)-methyltransferase non-catalytic subunit WDR4	−3.03	Phospho [S397(100)]
P81122	*Irs2*	Insulin receptor substrate 2	−3.03	Phospho [S66(97.6)]
Q8C0T5	*Sipa1l1*	Signal-induced proliferation-associated 1-like protein 1	−3.13	Phospho [S1528(100); S1624(100); S1626(100); S1629(99.1)]
P97868	*Rbbp6*	E3 ubiquitin-protein ligase RBBP6	−3.23	Phospho [S1179(100); S1329(100); S1644(98.4); S1646(98.4); S1651(100)]
E9Q4F7	*Ankrd11*	Ankyrin repeat domain-containing protein 11	−3.33	Phospho [S1070(100); S1832(99); S1844(100)]
Q8BG09	*Tmem184b*	Transmembrane protein 184B	−3.41	Phospho [S402(100); S403(100)]
P97496	*Smarcc1*	SWI/SNF complex subunit SMARCC1	−3.50	Phospho [S327(100); S329(100)]
Q80Y17	*Llgl1*	Lethal(2) giant larvae protein homolog 1	−3.65	Phospho [S982(100); S986(100); S989(98.6)]
Q64127	*Trim24*	Transcription intermediary factor 1-alpha	−3.79	Phospho [S1026(100); S1029(100)]
Q8C8R3	*Ank2*	Ankyrin-2	−4.03	Phospho [S1699(100); S1700(100); S1703(100); S2824(100); S2827(99.4); S3362(100)]
Q99PM3	*Gtf2a1*	Transcription initiation factor IIA subunit 1	−4.19	Phospho [S318(100); S323(100)]
Q63918	*Sdpr*	Caveolae-associated protein 2	−4.28	Phospho [S203(100); S204(100); S218(100); S293(100); S359(100); S363(100); T368(100)]
Q3UMU9-2	*Hdgfrp2*	Isoform 2 of Hepatoma-derived growth factor-related protein 2	−4.28	Phospho [S365(100); S366(100); S627(100); S628(100); S638(100)]

## Data Availability

Data sharing not applicable.

## Supplementary Materials

The following are available online at https://www.mdpi.com/article/10.3390/ijms23021006/s1.

## References

[1] Bonomi C.G., De Lucia V., Mascolo A.P., Assogna M., Motta C., Scaricamazza E., Sallustio F., Mercuri N.B., Koch G., Martorana A. (2021). Brain energy metabolism and neurodegeneration: Hints from CSF lactate levels in dementias. Neurobiol. Aging.

[2] Harris J.J., Jolivet R., Attwell D. (2012). Synaptic energy use and supply. Neuron.

[3] Uemura E., Greenlee H.W. (2006). Insulin regulates neuronal glucose uptake by promoting translocation of glucose transporter GLUT3. Exp. Neurol..

[4] Al-Zubaidi A., Heldmann M., Mertins A., Brabant G., Nolde J.M., Jauch-Chara K., Münte T.F. (2019). Impact of Hunger, Satiety, and Oral Glucose on the Association Between Insulin and Resting-State Human Brain Activity. Front. Hum. Neurosci..

[5] Kleinridders A., Ferris H.A., Cai W., Kahn C.R. (2014). Insulin action in brain regulates systemic metabolism and brain function. Diabetes.

[6] Fetterly T.L., Oginsky M.F., Nieto A.M., Alonso-Caraballo Y., Santana-Rodriguez Z., Ferrario C.R. (2021). Insulin Bidirectionally Alters NAc Glutamatergic Transmission: Interactions between Insulin Receptor Activation, Endogenous Opioids, and Glutamate Release. J. Neurosci..

[7] Huang C.-C., Lee C.-C., Hsu K.-S. (2004). An investigation into signal transduction mechanisms involved in insulin-induced long-term depression in the CA1 region of the hippocampus. J. Neurochem..

[8] Van der Heide L.P., Kamal A., Artola A., Gispen W.H., Ramakers G.M.J. (2005). Insulin modulates hippocampal activity-dependent synaptic plasticity in a N-methyl-d-aspartate receptor and phosphatidyl-inositol-3-kinase-dependent manner. J. Neurochem..

[9] Labouèbe G., Liu S., Dias C., Zou H., Wong J., Karunakaran S., Clee S., Phillips A.G., Boutrel B., Borgland S.L. (2013). Insulin induces long-term depression of ventral tegmental area dopamine neurons via endocannabinoids. Nat. Neurosci..

[10] Moult P.R., Harvey J. (2008). Hormonal regulation of hippocampal dendritic morphology and synaptic plasticity. Cell Adhes. Migr..

[11] Forny-Germano L., De Felice F.G., Vieira M.N.D.N. (2019). The Role of Leptin and Adiponectin in Obesity-Associated Cognitive Decline and Alzheimer’s Disease. Front. Neurosci..

[12] Takechi R., Galloway S., Pallebage-Gamarallage M.M., Wellington C.L., Johnsen R.D., Dhaliwal S.S., Mamo J.C. (2010). Differential effects of dietary fatty acids on the cerebral distribution of plasma-derived apo B lipoproteins with amyloid-beta. Br. J. Nutr..

[13] Barber T.M., Kyrou I., Randeva H.S., Weickert M.O. (2021). Mechanisms of Insulin Resistance at the Crossroad of Obesity with Associated Metabolic Abnormalities and Cognitive Dysfunction. Int. J. Mol. Sci..

[14] Solinas G., Becattini B. (2016). JNK at the crossroad of obesity, insulin resistance, and cell stress response. Mol. Metab..

[15] Melo H.M., Silva G.D.S.S.D., Sant’Ana M.R., Teixeira C.V.L., Clarke J.R., Coreixas V.S.M., de Melo B.C., Fortuna J.T., Forny-Germano L., Ledo J.H. (2020). Palmitate Is Increased in the Cerebrospinal Fluid of Humans with Obesity and Induces Memory Impairment in Mice via Pro-inflammatory TNF-α. Cell Rep..

[16] Karmi A., Iozzo P., Viljanen A., Hirvonen J., Fielding B.A., Virtanen K., Oikonen V., Kemppainen J., Viljanen T., Guiducci L. (2010). Increased brain Fatty Acid Uptake in Metabolic Syndrome. Diabetes.

[17] Hernandez-Caceres M.P., Toledo-Valenzuela L., Diaz-Castro F., Avalos Y., Burgos P., Narro C., Pena-Oyarzun D., Espinoza-Caicedo J., Cifuentes-Araneda F., Navarro-Aguad F. (2019). Palmitic Acid Reduces the Autophagic Flux and Insulin Sensitivity Through the Activation of the Free Fatty Acid Receptor 1 (FFAR1) in the Hypothalamic Neuronal Cell Line N43/5. Front. Endocrinol..

[18] Mayer C.M., Belsham D.D. (2010). Palmitate attenuates Insulin Signaling and Induces Endoplasmic Reticulum Stress and Apoptosis in Hypothalamic Neurons: Rescue of Resistance and Apoptosis through Adenosine 5′ Monophosphate-Activated Protein Kinase Activation. Endocrinology.

[19] Tse E.K., Salehi A., Clemenzi M.N., Belsham D.D. (2018). Role of the saturated fatty acid palmitate in the interconnected hypothalamic control of energy homeostasis and biological rhythms. Am. J. Physiol. Metab..

[20] Lemmer I.L., Willemsen N., Hilal N., Bartelt A. (2021). A guide to understanding endoplasmic reticulum stress in metabolic disorders. Mol. Metab..

[21] Kratz M., Coats B.R., Hisert K.B., Hagman D., Mutskov V., Peris E., Schoenfelt K.Q., Kuzma J.N., Larson I., Billing P.S. (2014). Metabolic Dysfunction Drives a Mechanistically Distinct Proinflammatory Phenotype in Adipose Tissue Macrophages. Cell Metab..

[22] Zand H., Morshedzadeh N., Naghashian F. (2017). Signaling pathways linking inflammation to insulin resistance. Diabetes Metab. Syndr..

[23] Liu C., Feng X., Li Q., Wang Y., Li Q., Hua M. (2016). Adiponectin, TNF-α and inflammatory cytokines and risk of type 2 diabetes: A systematic review and meta-analysis. Cytokine.

[24] Moller D.E. (2000). Potential Role of TNF-α in the Pathogenesis of Insulin Resistance and Type 2 Diabetes. Trends Endocrinol. Metab..

[25] Yaribeygi H., Farrokhi F.R., Butler A.E., Sahebkar A. (2018). Insulin resistance: Review of the underlying molecular mechanisms. J. Cell. Physiol..

[26] Luquet S., Magnan C. (2009). The central nervous system at the core of the regulation of energy homeostasis. Front. Biosci. (Sch. Ed.).

[27] De Luca C., Olefsky J.M. (2008). Inflammation and insulin resistance. FEBS Lett..

[28] Van Gennip A.C., Stehouwer C.D., van Boxtel M.P., Verhey F.R., Koster A., Kroon A.A., Köhler S., van Greevenbroek M.M., Wesselius A., Eussen S.J. (2021). Association of Type 2 Diabetes, According to the Number of Risk Factors Within Target Range, With Structural Brain Abnormalities, Cognitive Performance, and Risk of Dementia. Diabetes Care.

[29] Whitmer R.A., Karter A.J., Yaffe K., Quesenberry C.P., Selby J.V. (2009). Hypoglycemic episodes and risk of dementia in older patients with type 2 diabetes mellitus. JAMA.

[30] Deng W., Aimone J.B., Gage F.H. (2010). New neurons and new memories: How does adult hippocampal neurogenesis affect learning and memory?. Nat. Rev. Neurosci..

[31] Maguire E.A., Frackowiak R., Frith C. (1996). Learning to find your way: A role for the human hippocampal formation. Proc. Biol. Sci..

[32] Verret L., Mann E., Hang G.B., Barth A.M., Cobos I., Ho K., Devidze N., Masliah E., Kreitzer A.C., Mody I. (2012). Inhibitory Interneuron Deficit Links Altered Network Activity and Cognitive Dysfunction in Alzheimer Model. Cell.

[33] Jacobs D., Sano M., Marder K., Bell K., Bylsma F., Lafleche G., Albert M., Brandt J., Stern Y. (1994). Age at onset of Alzheimer’s disease: Relation to pattern of cognitive dysfunction and rate of decline. Neurology.

[34] O’Kusky J.R., Ye P., D’Ercole A.J. (2000). Insulin-Like Growth Factor-I Promotes Neurogenesis and Synaptogenesis in the Hippocampal Dentate Gyrus during Postnatal Development. J. Neurosci..

[35] Biessels G.J., Reagan L.P. (2015). Hippocampal insulin resistance and cognitive dysfunction. Nat. Rev. Neurosci..

[36] Leinninger G.M., Russell J.W., Van Golen C.M., Berent A., Feldman E.L. (2004). Insulin-like growth factor-I regulates glucose-induced mitochondrial depolarization and apoptosis in human neuroblastoma. Cell Death Differ..

[37] Waly M., Olteanu H., Banerjee R., Choi S.-W., Mason J.B., Parker B., Sukumar S., Shim S., Sharma A., Benzecry J.M. (2004). Activation of methionine synthase by insulin-like growth factor-1 and dopamine: A target for neurodevelopmental toxins and thimerosal. Mol. Psychiatry.

[38] Tanno B., Mancini C., Vitali R., Mancuso M., McDowell H.P., Dominici C., Raschellà G. (2006). Down-Regulation of Insulin-Like Growth Factor I Receptor Activity by NVP-AEW541 Has an Antitumor Effect on Neuroblastoma Cells In vitro and In vivo. Clin. Cancer Res..

[39] Nieto-Estévez V., Defterali Ç., Vicario-Abejón C. (2016). IGF-I: A key growth factor that regulates neurogenesis and synaptogenesis from embryonic to adult stages of the brain. Front. Neurosci..

[40] Otaegi G., Yusta-Boyo M.J., Vergaño-Vera E., Méndez-Gómez H.R., Carrera A., Abad J.L., González M., de la Rosa E.J., Vicario-Abejón C., de Pablo F. (2006). Modulation of the PI 3-kinase–Akt signalling pathway by IGF-I and PTEN regulates the differentiation of neural stem/precursor cells. J. Cell Sci..

[41] Spinelli M., Fusco S., Grassi C. (2019). Brain Insulin Resistance and Hippocampal Plasticity: Mechanisms and Biomarkers of Cognitive Decline. Front. Neurosci..

[42] Winocur G., Greenwood C.E., Piroli G.G., Grillo C., Reznikov L., Reagan L.P., McEwen B.S. (2005). Memory impairment in obese Zucker rats: An investigation of cognitive function in an animal model of insulin resistance and obesity. Behav. Neurosci..

[43] Rahman S.O., Panda B.P., Parvez S., Kaundal M., Hussain S., Akhtar M., Najmi A.K. (2019). Neuroprotective role of astaxanthin in hippocampal insulin resistance induced by Abeta peptides in animal model of Alzheimer’s disease. Biomed. Pharmacother..

[44] Akhtar A., Bishnoi M., Sah S.P. (2020). Sodium orthovanadate improves learning and memory in intracerebroventricular-streptozotocin rat model of Alzheimer’s disease through modulation of brain insulin resistance induced tau pathology. Brain Res. Bull..

[45] Ho N., Sommers M.S., Lucki I. (2013). Effects of diabetes on hippocampal neurogenesis: Links to cognition and depression. Neurosci. Biobehav. Rev..

[46] Nagamatsu S., Nakamichi Y., Yamamura C., Matsushima S., Watanabe T., Ozawa S., Furukawa H., Ishida H. (1999). Decreased expression of t-SNARE, syntaxin 1, and SNAP-25 in pancreatic beta-cells is involved in impaired insulin secretion from diabetic GK rat islets: Restoration of decreased t-SNARE proteins improves impaired insulin secretion. Diabetes.

[47] Garcia-Serrano A.M., Duarte J.M.N. (2020). Brain Metabolism Alterations in Type 2 Diabetes: What Did We Learn from Diet-Induced Diabetes Models?. Front. Neurosci..

[48] Kim M.-H., Choi J., Yang J., Chung W., Kim J.-H., Paik S.K., Kim K., Han S., Won H., Bae Y.-S. (2009). Enhanced NMDA Receptor-Mediated Synaptic Transmission, Enhanced Long-Term Potentiation, and Impaired Learning and Memory in Mice Lacking IRSp53. J. Neurosci..

[49] Nowak F.V. (2018). Porf-2 = Arhgap39 = Vilse: A Pivotal Role in Neurodevelopment, Learning and Memory. eNeuro.

[50] Sibson N.R., Dhankhar A., Mason G.F., Rothman D.L., Behar K., Shulman R.G. (1998). Stoichiometric coupling of brain glucose metabolism and glutamatergic neuronal activity. Proc. Natl. Acad. Sci. USA.

[51] Macauley S.L., Stanley M., Caesar E.E., Yamada S.A., Raichle M.E., Perez R., Mahan T.E., Sutphen C.L., Holtzman D.M. (2015). Hyperglycemia modulates extracellular amyloid-beta concentrations and neuronal activity in vivo. J. Clin. Investig..

[52] Rocher A.B., Chapon F., Blaizot X., Baron J.-C., Chavoix C. (2003). Resting-state brain glucose utilization as measured by PET is directly related to regional synaptophysin levels: A study in baboons. NeuroImage.

[53] Mosconi L. (2005). Brain glucose metabolism in the early and specific diagnosis of Alzheimer’s disease. FDG-PET studies in MCI and AD. Eur. J. Nucl. Med. Mol. Imaging.

[54] Baker L.D., Cross D., Minoshima S., Belongia D., Watson G.S., Craft S. (2011). Insulin Resistance and Alzheimer-like Reductions in Regional Cerebral Glucose Metabolism for Cognitively Normal Adults with Prediabetes or Early Type 2 Diabetes. Arch. Neurol..

[55] Anchisi D., Borroni B., Franceschi M., Kerrouche N., Kalbe E., Beuthien-Beumann B., Cappa S., Lenz O., Ludecke S., Marcone A. (2005). Heterogeneity of Brain Glucose Metabolism in Mild Cognitive Impairment and Clinical Progression to Alzheimer Disease. Arch. Neurol..

[56] Rojas S., Herance J.R., Gispert J.D., Abad S., Torrent É., Jiménez X., Pareto D., Perpiña U., Sarroca S., Rodríguez E. (2013). In vivo evaluation of amyloid deposition and brain glucose metabolism of 5XFAD mice using positron emission tomography. Neurobiol. Aging.

[57] Parihar M.S., Brewer G.J. (2010). Amyloid-beta as a modulator of synaptic plasticity. J. Alzheimers Dis..

[58] Gaddam M., Singh A., Jain N., Avanthika C., Jhaveri S., De la Hoz I., Sanka S., Goli S.R. (2021). A Comprehensive Review of Intranasal Insulin and Its Effect on the Cognitive Function of Diabetics. Cureus.

[59] Torabi N., Noursadeghi E., Shayanfar F., Nazari M., Fahanik-Babaei J., Saghiri R., Khodagholi F., Eliassi A. (2021). Intranasal insulin improves the structure–function of the brain mitochondrial ATP–sensitive Ca2+ activated potassium channel and respiratory chain activities under diabetic conditions. Biochim. Biophys. Acta (BBA)—Mol. Basis Dis..

[60] Jamal S., Ali W., Nagpal P., Grover A., Grover S. (2021). Predicting phosphorylation sites using machine learning by integrating the sequence, structure, and functional information of proteins. J. Transl. Med..

[61] Alexovič M., Urban P.L., Tabani H., Sabo J. (2020). Recent advances in robotic protein sample preparation for clinical analysis and other biomedical applications. Clin. Chim. Acta.

[62] Garcia T.G., Poncet S., Derouiche A., Shi L., Mijakovic I., Noirot-Gros M.-F. (2016). Role of Protein Phosphorylation in the Regulation of Cell Cycle and DNA-Related Processes in Bacteria. Front. Microbiol..

[63] Baker N., Catta-Preta C.M.C., Neish R., Sadlova J., Powell B., Alves-Ferreira E.V.C., Geoghegan V., Carnielli J.B.T., Newling K., Hughes C. (2021). Systematic functional analysis of Leishmania protein kinases identifies regulators of differentiation or survival. Nat. Commun..

[64] Chen Y., Yuan J. (2021). The post translational modification of key regulators of ATR signaling in DNA replication. Genome Instab. Dis..

[65] Barford D., Das A.K., Egloff M.-P. (1998). The structure and mechanism of protein phosphatases: Insights into catalysis and regulation. Annu. Rev. Biophys. Biomol. Struct..

[66] Ubersax J.A., Ferrell J.E. (2007). Mechanisms of specificity in protein phosphorylation. Nat. Rev. Mol. Cell Biol..

[67] Krebs E.G., Beavo J.A. (1979). Phosphorylation-Dephosphorylation of Enzymes. Annu. Rev. Biochem..

[68] Cohen P. (2002). The origins of protein phosphorylation. Nat. Cell Biol..

[69] Litichevskiy L., Peckner R., Abelin J.G., Asiedu J.K., Creech A.L., Davis J.F., Davison D., Dunning C.M., Egertson J.D., Egri S. (2018). A Library of Phosphoproteomic and Chromatin Signatures for Characterizing Cellular Responses to Drug Perturbations. Cell Syst..

[70] Chen X., Wei S., Ji Y., Guo X., Yang F. (2015). Quantitative proteomics using SILAC: Principles, applications, and developments. Proteomics.

[71] Yoon J.H., Kim D., Jang J.-H., Ghim J., Park S., Song P., Kwon Y., Kim J., Hwang D., Bae Y.-S. (2015). Proteomic Analysis of the Palmitate-induced Myotube Secretome Reveals Involvement of the Annexin A1-Formyl Peptide Receptor 2 (FPR2) Pathway in Insulin Resistance. Mol. Cell. Proteom..

[72] Yoon J.H., Song P., Jang J.-H., Kim D.-K., Choi S., Kim J., Ghim J., Kim D., Park S., Lee H. (2011). Proteomic Analysis of Tumor Necrosis Factor-Alpha (TNF-α)-Induced L6 Myotube Secretome Reveals Novel TNF-α-Dependent Myokines in Diabetic Skeletal Muscle. J. Proteome Res..

[73] Cheyuo C., Aziz M., Wang P. (2019). Neurogenesis in Neurodegenerative Diseases: Role of MFG-E8. Front. Neurosci..

[74] Wennstrom M., Nielsen H.M. (2012). Cell adhesion molecules in Alzheimer’s disease. Degener. Neurol. Neuromuscul. Dis..

[75] Bozzo C., Graziola F., Chiocchetti A., Canonico P.L. (2010). Estrogen and beta-amyloid toxicity: Role of integrin and PI3-K. Mol. Cell Neurosci..

[76] Rowan M.J., Klyubin I., Wang Q., Hu N.W., Anwyl R. (2007). Synaptic memory mechanisms: Alzheimer’s disease amyloid beta-peptide-induced dysfunction. Biochem. Soc. Trans..

[77] Akiyama H., McGeer P.L. (1990). Brain microglia constitutively express beta-2 integrins. J. Neuroimmunol..

[78] Wang P., Ye Y. (2021). Filamentous recombinant human Tau activates primary astrocytes via an integrin receptor complex. Nat. Commun..

[79] Hardie D.G. (2011). AMP-activated protein kinase—An energy sensor that regulates all aspects of cell function. Genes Dev..

[80] Chen M., Huang N., Liu J., Huang J., Shi J., Jin F. (2021). AMPK: A bridge between diabetes mellitus and Alzheimer’s disease. Behav. Brain Res..

[81] Lu J., Wu D.-M., Zheng Y.-L., Hu B., Zhang Z.-F., Shan Q., Zheng Z.-H., Liu C.-M., Wang Y.-J. (2010). Quercetin activates AMP-activated protein kinase by reducing PP2C expression protecting old mouse brain against high cholesterol-induced neurotoxicity. J. Pathol..

[82] Gupta A., Bisht B., Dey C.S. (2011). Peripheral insulin-sensitizer drug metformin ameliorates neuronal insulin resistance and Alzheimer’s-like changes. Neuropharmacology.

[83] Seixas da Silva G.S., Melo H.M., Lourenco M.V., Lyra E.S.N.M., de Carvalho M.B., Alves-Leon S.V., de Souza J.M., Klein W.L., da-Silva W.S., Ferreira S.T. (2017). Amyloid-beta oligomers transiently inhibit AMP-activated kinase and cause metabolic defects in hippocampal neurons. J. Biol. Chem..

[84] Julien C., Tremblay C., Émond V., Lebbadi M., Salem N., Bennett D.A., Calon F. (2009). Sirtuin 1 Reduction Parallels the Accumulation of Tau in Alzheimer Disease. J. Neuropathol. Exp. Neurol..

[85] Xu Y., Hu R., He D., Zhou G., Wu H., Xu C., He B., Wu L., Wang Y., Chang Y. (2020). Bisdemethoxycurcumin inhibits oxidative stress and antagonizes Alzheimer’s disease by up-regulating SIRT1. Brain Behav..

